# A Simple Self-Maintaining Metabolic System: Robustness, Autocatalysis, Bistability

**DOI:** 10.1371/journal.pcbi.1000872

**Published:** 2010-08-05

**Authors:** Gabriel Piedrafita, Francisco Montero, Federico Morán, María Luz Cárdenas, Athel Cornish-Bowden

**Affiliations:** 1 Departamento de Bioquímica y Biología Molecular I, Facultad de Ciencias Químicas, Universidad Complutense de Madrid, Madrid, Spain; 2 Bioénergétique et Ingénierie des Protéines, Centre National de la Recherche Scientifique, Marseille, France; ETH Zurich, Switzerland

## Abstract

A living organism must not only organize itself from within; it must also maintain its organization in the face of changes in its environment and degradation of its components. We show here that a simple (M,R)-system consisting of three interlocking catalytic cycles, with every catalyst produced by the system itself, can both establish a non-trivial steady state and maintain this despite continuous loss of the catalysts by irreversible degradation. As long as at least one catalyst is present at a sufficient concentration in the initial state, the others can be produced and maintained. The system shows bistability, because if the amount of catalyst in the initial state is insufficient to reach the non-trivial steady state the system collapses to a trivial steady state in which all fluxes are zero. It is also robust, because if one catalyst is catastrophically lost when the system is in steady state it can recreate the same state. There are three elementary flux modes, but none of them is an enzyme-maintaining mode, the entire network being necessary to maintain the two catalysts.

## Author Summary

The question of whether a whole organism (as opposed to particular properties of an organism) can be modeled in the computer has been controversial. As a step towards resolving it, we have studied the feasibility of simulating the behavior of a simple theoretical model in which all the catalysts needed for the metabolism of a system are themselves products of the metabolism itself, and in which there is a continuous loss of catalysts in unavoidable degradation reactions. In addition to a trivial (“dead”) steady state in which all rates are zero, the model is capable of establishing a stable non-trivial steady state with finite and reproducible fluxes. This can be achieved by “seeding” it with a sufficient quantity of at least one of the catalysts needed for functioning. It is also robust, because it can recover from a catastrophic disappearance of a catalyst.

## Introduction 

Several theories of life [1]–[5] coincide in the importance that they give to *metabolic closure*, the necessity for all of the catalysts essential for survival of an organism to be produced internally, as an organism cannot rely on any external agent for maintaining it. The same considerations must apply to the self-maintaining systems at the origin of life [6], [7]. Rosen [1] expressed this idea that catalysts must be produced by the system itself by saying that it must be *closed to efficient causation*. These theories differ in their details, and each includes important points missing from the others. Among them the theory of (M,R)-system s, or *metabolism–replacement systems*, perhaps comes closest to a complete explanation of life, but it is usually presented in abstract terms [1] that make it difficult to relate it to any ordinary ideas of chemistry, metabolism and catalysis.

**Figure 1:**
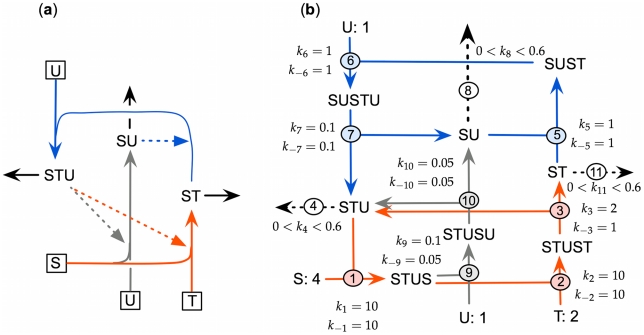
A model of an (M,R)-system. (a) The metabolites shown inside squares (input) are considered to be “external” and to have fixed concentrations. The reactions shown in red constitute the metabolic process, those in blue the replacement process, and in gray the replacement of the replacement catalyst. (b) Expanded version of the model in which each catalyzed reaction is expanded into a cycle of three chemical reactions with explicit rate constants. Each forward rate constant refers to the reaction in the direction of the arrow, and the three degradation reactions, steps 4, 8 and 11, are assumed to be uncatalyzed and irreversible. All rate constants are treated as constant with the values shown, apart from 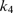, 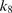 and 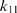, which are varied (but kept equal to one another) in the range 0.0–0.6. The three external reactants S, T and U are assumed to have the constant concentrations shown. All other concentrations are variable. All units are arbitrary, but they are consistent (i.e. the same units of time and quantity of matter apply throughout) and the model can be written in dimensionless form, if desired. In addition, the numerical values assigned to the rate constants and external concentrations are also arbitrary.

To give concrete expression to the idea of an (M,R)-system , and to evaluate its possible relevance to the origin of life, we proposed [8]–[10] a simple system of three interlocking cycles: a *metabolic* process 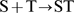 produces a metabolite ST from external precursors S and T in a reaction catalyzed by a component STU that is itself the product of a *replacement* process 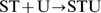, in which U is another external precursor. The replacement process is necessary because STU, as a biological molecule, cannot be assumed to have an infinite lifetime, and even if it did it would be diluted by growth of the system and by other processes. Moreover, replacement also needs a catalyst, which also needs to be replaced. To escape immediately from the implied infinite regress we proposed that replacement is catalyzed by a similar type of molecule, SU, that results from a secondary reaction catalyzed by STU, 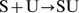. This system, illustrated in Fig. 1, is closed to efficient causation, because each of the three reactions is catalyzed by a product of the system itself. In our original proposal we assumed that only STU and SU were subject to unavoidable degradation (see Fig. 1b of [10]), but there is no logical reason to suppose that the other product of the system, ST, is indefinitely stable, especially as it is assumed to be a molecule similar to SU. In Fig. 1, therefore, there is a third degradation reaction, reaction 11.

A controversial aspect of Rosen's analysis is his contention that a system closed to efficient causation cannot have computable models [11]–[14]. Many aspects of biological systems can, of course, be simulated in the computer, and many examples of metabolic simulation can be found in the literature, but typically these examples do not simulate systems that are closed to efficient causation. In the recent simulation of aspartate metabolism in *Arabidopsis thaliana* [15], for example, the enzymes were taken as given; their production was not simulated. We discuss this controversy elsewhere [16] and will not do so here, apart from noting that there is no obvious reason why the system illustrated in Fig. 1 should not be simulated. On the contrary, it can certainly be simulated, as we shall show, with results that shed light of the conditions that need to be fulfilled by a self-maintaining system.

We shall show that a simple (M,R)-system can be robust, capable of a recovering from the loss of most of its catalysts, and in addition has the interesting property of bistability. As Delbrück [17] emphasized many years ago, multistability is also an important property for all but the simplest living organisms because it is essential for differentiation, an idea that has subsequently been developed by other authors [18]. Bistability can arise in systems considerably smaller and simpler [19] than the one we discuss in this paper, but we are concerned here with (M,R)-system s, which must be closed to efficient causation.

## Model

For the system to be simulated it needs to be defined in precise numerical terms, and for doing this it is convenient to expand the catalytic processes shown in Fig. 1a into the cycles of chemical reactions shown in Fig. 1b [9]. There is no fundamental difference in this model between catalysts (“enzymes”) and metabolites, and elsewhere [10] we have argued that no fundamental difference exists: all enzymes are products of the system in which they participate, and are thus metabolites, and many conventional metabolites (for example, ornithine in the urea cycle) participate in cycles of reactions, and thus satisfy the definition of a catalyst.

All simulations and studies of the stability of the steady states found were done with Matlab and checked with COPASI [20], or vice versa, and stoichiometric network analysis was done with MetaTool [21]. In the present paper all simulation is deterministic.

As we shall be supposing that the system in Fig. 1 can continue in operation indefinitely, despite containing irreversible degradation steps, we need to consider the thermodynamic feasibility of what we propose. In effect, we assume that the overall chemical reactions 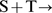 degradation products, 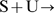 degradation products and 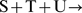 degradation products are irreversible, that synthesis of ST in the reaction 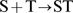 is thermodynamically favored, and that the concentrations of the external molecules S, T and U are constant, either because the quantities consumed by the system are too small to have any effect on their concentrations, or because they are buffered by external chemistry. External constraints on a system of chemical reactions can be applied in two main ways, either with constant external concentrations or with constant input fluxes. In this model we have chosen the former approach, primarily to facilitate comparison with earlier work [8]–[10].

In this context it is important to note that organizational closure does not imply thermodynamic closure, or vice versa. In the Aristotelean terminology favored by Rosen [1], closure to efficient causation is not the same as closure to material causation [10]. An organism must clearly be open to material causation — it “feeds on negative entropy”, in Schrödinger's words [22] — but it can still synthesize all of its catalysts, and thus be closed to efficient causation. In a third type of closure, independent from both of these, an individual organism must be structurally closed, separated from other individuals by a skin or other barrier. This aspect was given almost no attention by Rosen [1], and we shall not discuss it further here, but it is clearly necessary, and it forms an important element of other theories of life such as autopoiesis [3].

## Results

### Stationary solutions and self-maintenance of the (M,R) system

The concentration evolution of the different metabolites in Fig. 1b can be described by a series of ordinary differential equations:
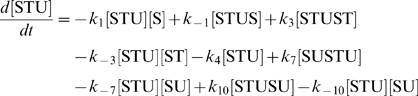


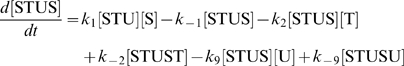


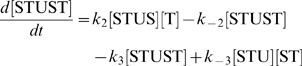


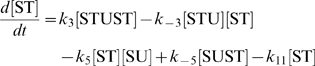


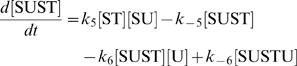


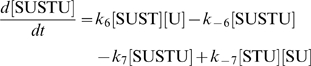


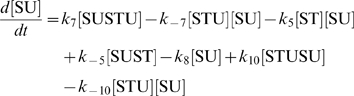


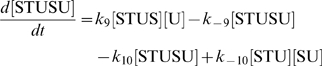
The simple non-linear terms in these equations arise from applying simple mass action kinetics to the bimolecular steps.

Stationary solutions of the system of Fig. 1b were obtained by two different methods, numerical integration of the previous set of differential equations, and analytical solution of the nonlinear algebraic equations. Both revealed the existence of a region with three distinct steady states, one trivial and two non-trivial. It is obvious that the system shown in Fig. 1 cannot undergo any reactions if no form of any catalyst is present. Less obvious is whether it can construct itself and maintain itself indefinitely if it is seeded with a sufficient quantity of one catalyst. This has been tested in the first instance with various values in the range 0–0.6 of the degradation rate constants 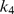, 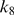 and 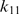, other rate constants as defined in Fig. 1, and various initial concentrations of one intermediate, STU, all other intermediate concentrations being set initially to zero.

**Figure 2:**
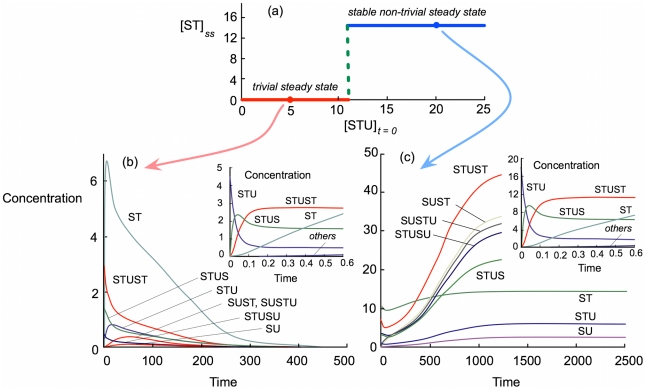
Steady states reached with the model. The model was simulated for 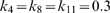 and various values of 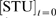, the initial concentration of STU, as shown, and allowed to evolve until a steady state was reached. (a) For 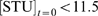 the trivial steady state was always reached, whereas for 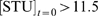 the non-trivial stable steady state was reached. (b) The evolution from the red point in panel (a), with 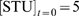 is shown. The behavior at very short times is illustrated in the inset. (c) The evolution from the blue point in panel (a), with 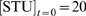, is shown. The behavior at very short times is illustrated in the inset. Note that the two insets are qualitatively very similar to one another, but the long-term trends in (b) and (c) are very different.

For 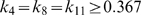 the system cannot construct itself or maintain itself despite seeding with large or small initial concentrations of STU, and it always ends in a trivial steady state with all concentrations and all rates zero. However, with smaller degradation rate constants it can reach either the trivial steady state or a non-trivial steady state with all concentrations and rates non-zero, i.e. a self-maintaining regime. The results are summarized in Fig. 2 for 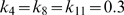 and different initial concentrations of STU. For 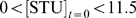 the system reached a trivial steady state with all concentrations zero, but at any 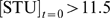 it reached a non-trivial steady state with 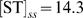 and all other concentrations and all rates non-zero. Hence there is a none-to-all transition at this critical point, as indicated by the broken line in Fig. 2a.

STU is not of course the only catalytic intermediate that could be used for seeding the system, and results with each of the others, and for some pairs of intermediates, are shown in Table 1, for two values of 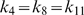. Two important points are evident in this table: first, any metabolite apart from ST or SU can be separately used to seed the system, and although the concentration of seed metabolite necessary to drive it to a non-trivial steady state varies with the seed, the steady state reached depends only on the degradation rate constants, and is independent of the identity of the seed. We have also made simulations with various mixtures of metabolites used as seeds and these generalizations remain true.

**Table 1:** Non-trivial steady states reached from different seed metabolites.

Seed	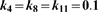		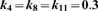	
	Minimum initial concentration	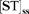	Minimum initial concentration	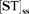
STU	0.135	15.63	11.460	14.32
STUS	0.135	15.63	11.374	14.32
STUST	0.135	15.63	11.378	14.32
ST	—	no steady state	—	no steady state
SU	—	no steady state	—	no steady state
SUST	0.355	15.63	9.896	14.32
SUSTU	0.278	15.63	8.845	14.32
STUSU	0.114	15.63	6.801	14.32
STU+SU	0.099	15.63	5.251	14.32
ST+SUST	0.295	15.63	8.143	14.32
SU+STUS	0.099	15.63	5.184	14.32
ST+SU	0.455	15.63	10.433	14.32

The reason why ST and SU cannot act as seed can be seen by inspection of Fig. 1b: neither of these metabolites reacts directly with any of the external reactants S, T and U, and so no reaction can take place if none of the other metabolites are present. However, ST and SU can react with one another to give a product SUST capable of participating in additional reactions and closing all the loops. Not surprisingly therefore, the system can be seeded with a mixture of ST and SU even though neither of them is effective alone.

### Bistability and hysteretic behavior

**Figure 3:**
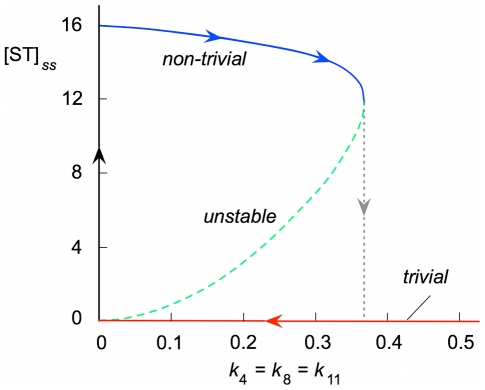
Bifurcation plot. For 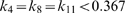 there is a region of bistability in which both trivial and non-trivial stable steady states are separated by an unstable steady state. When the system is at equilibrium with 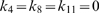, the only possible stable steady state is the non-trivial steady state with [ST] = 16, as indicated by the arrows. If the decay constants are increased (proceeding to the right in the plot), the system remains in a non-trivial steady state until it falls abruptly to zero — the trivial steady state — exactly when leaving the bistability region. However, when starting from these final conditions, with every concentration zero, the initial trajectory cannot be reversed, because the system cannot “climb” to the non-trivial steady state until it is close to the equilibrium condition (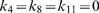). Only when approaching this condition can it experience a large jump after the appearance of small fluctuations in the concentrations. In brief, the direction of movement determines the specific behavior: the jump is detected at 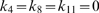 when going to the left and at 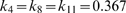 when going to the right.

To verify the stability of the steady states, the Jacobian matrices were evaluated at the steady states obtained, and the eigenvectors and eigenvalues calculated. For those conditions in which three steady states were obtained, 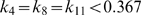, the trivial and one of the non-trivial solutions always have all eigenvalues with negative real parts, and thus are asymptotically stable. Obviously, they correspond to those reached by numerical integration experiments. The additional non-trivial steady state calculated by the analytical solution of the non-linear algebraic equations has, however, one of the eigenvalues with positive real part, and is therefore an unstable steady state (a saddle point), so in this region the system exhibits bistability. Beyond the critical value, 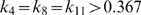, only the trivial steady solution exists and is asymptotically stable, i.e. each of its eigenvalues has a negative real part. These results are summarized in the bifurcation diagram illustrated in Fig. 3.

The diagram of Fig. 3 predicts a sort of hysteretic behavior: if the system is in the stable non-trivial steady state with small values of the decay rate constants, it remains in the same state when these constants are increased, until it abruptly collapses to the trivial steady state when the critical point 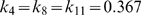 is reached. Once in the trivial steady state, it remains there even when the decay rate constants are decreased below the critical point. The hysteretic cycle cannot be completed unless we allow the possible appearance of trace quantities of any intermediate (such as might result from external chemistry) that could allow the system to recover the non-trivial steady state when close enough to the equilibrium condition 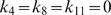.

**Figure 4:**
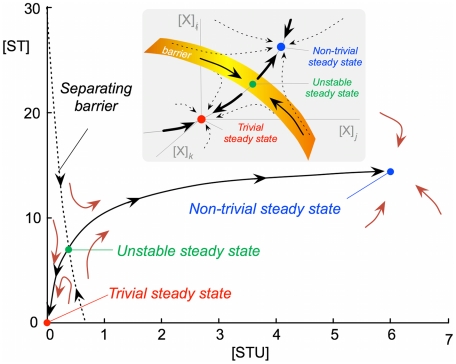
Planar section of the multidimensional phase diagram. The calculation refers to 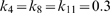. The point shown in green corresponds to the unstable steady state, which is contained in a barrier separating the attraction areas of the trivial steady state (point shown in red) and the non-trivial steady state (point shown in blue). The brown arrows represent a schematic illustration of the orbits followed in approaching the steady states. The inset illustrates schematically that the main plot is a two-dimensional slice of a multidimensional reality.

**Figure 5:**
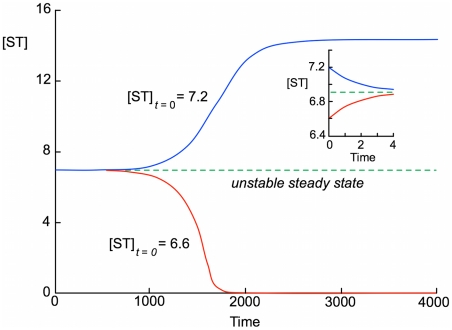
Time evolution from starting points close to the unstable steady state. Simulations were done with 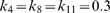. The initial concentration of ST was 6.6 (red curve) or 7.2 (blue curve), and other concentrations were set to those in the unstable steady state. The inset shows the time dependences at very low times, which are in the opposite directions from the long-term trends.

The unstable steady state that appears in those conditions of bistability, 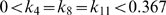, belongs to a separating barrier that constitutes a hypersurface limiting the attractor regions of both trivial and non-trivial stable steady states. A planar region of the phase diagram is illustrated in Fig. 4 for 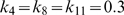. Different initial conditions close enough to the separating barrier could drive the system either to one stable steady state or the other, as shown in Fig. 5.

### Robustness of the stable non-trivial steady state

**Figure 6:**
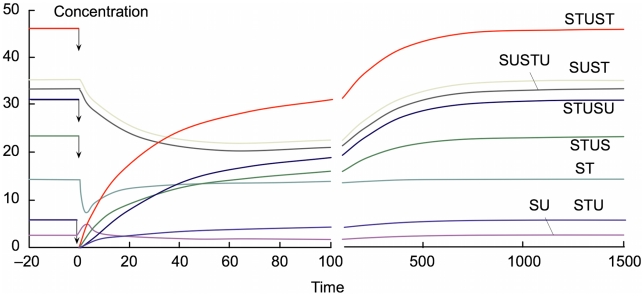
Recovery from a catastrophic loss of catalyst. The figure shows the time evolution of the system, starting from the stable non-trivial steady state for 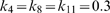 after the concentrations of all forms of STU (i.e. not only STU itself but also STUS, STUST and STUSU) are abruptly set to zero, as indicated by the arrows at time zero (leaving the others at their values in the non-trivial steady state).

It is clear that the system as described is capable of reaching a stable non-trivial steady state with finite fluxes and finite concentrations of all intermediates. However, before it can be regarded as a useful model of a self-maintaining system, and thus relevant to the early stages of metabolic evolution, it needs to be shown to be capable of recovering from catastrophic loss of one or more catalysts. To test this, it was allowed to reach the non-trivial stable steady state characteristic of 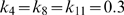, and the concentrations of all forms of STU (not only STU itself but also STUS, STUST and STUSU) were then abruptly set to zero, the others being left at their steady-state values. As seen in Fig. 6, both intermediate concentrations return to the previous steady-state values.

As STU catalyzes two different processes (synthesis both of SU and of ST), loss of STU is clearly the most stringent loss of catalyst one could consider, but for completeness we also tested the effect of loss of all forms of SU, with similar results. All of this shows that the system is highly robust, not only for infinitesimal perturbations, as tested by analysis of the Jacobian matrix, but also for large perturbations. Unless it is perturbed to such a large extent that the separating barrier mentioned is crossed, e.g. below the threshold requirements listed in Table 1 (for individual metabolites, but generalizable to combinations of metabolites), it always returns to the same non-trivial steady state. It can equally resist very large increases in metabolite concentrations, for example, when ST was abruptly raised to 100 times its steady-state value the system returned rapidly to the same steady state.

### Stoichiometric network analysis

**Figure 7:**
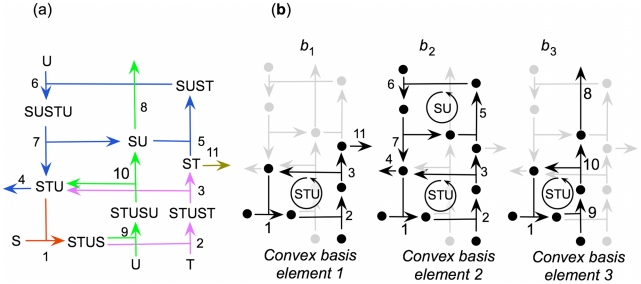
Stoichiometric analysis of the model. (a) The model contains five reaction subsets, consisting of reaction 1 (red), reactions 2 and 3 (magenta), reactions 4, 5, 6 and 7 (blue), reactions 8, 9 and 10 (green); and reaction 11 (gray). (b) There are three elements of the basis, one consisting of reactions 1, 2, 3 and 11 (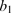) and thus corresponding to the metabolic pathway, a second consisting of reactions 1, 2, 3, 4, 5, 6 and 7 (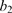), corresponding to both metabolic and replacement cycles, and the last consisting of reactions 1, 8, 9 and 10 (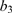), which is the pathway that replaces the replacement catalyst SU. Note that elements 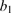 and 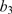 do not produce STU, and element 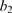 produces neither SU nor ST, each of which is produced by the other two elements. Thus none of these elements is an enzyme-maintaining mode [23].

With the use of MetaTool [21] we have analyzed the structure of the model by means of an approximation to a stoichiometric analysis in the steady state. In this analysis. S, T and U are considered as external metabolites, the others being considered internal. With the rates 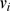 numbered as in Fig. 1b the reaction subsets 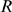 are as follows:
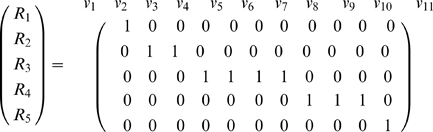
As seen in this equation, subsets of reactions operate at the same rate in the steady state, i.e. 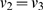, 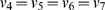 and 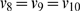, as illustrated in Fig. 7a. Notice that the degradation rates 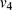 and 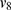 for the two catalysts STU and SU are in the same subsets as the corresponding replacement reactions: 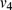 with 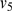, 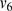 and 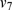; but 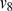 with 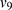 and 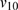. This explains how the replacement can efficiently balance the decay of each catalyst in the steady state.

The resulting convex basis can be expressed in the following way:
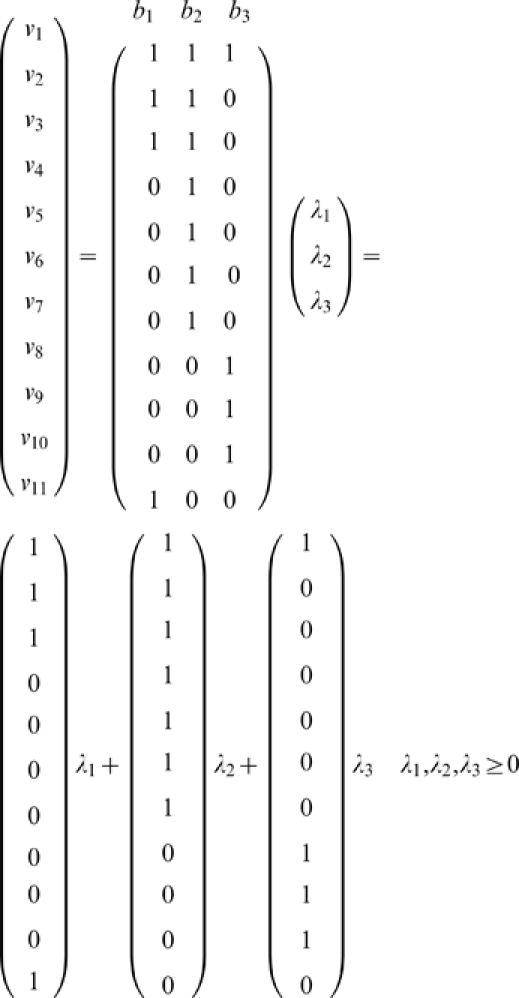
All three basis elements are shown schematically in Fig. 7b. The first, 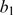, includes the reactions involved in the metabolism process, 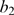 corresponds to both the metabolic and replacement cycles, and the third, 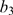, is the pathway that replaces the replacement catalyst SU. As previously shown in the subsets of reactions, the rate 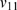 of decay of ST does not share the same rate with any other reaction. However, it also is compensated as a consequence of the performance of the metabolic reactions 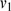, 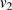 and 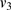, as deduced from the inspection of the first element of the convex basis.

**Figure 8:**
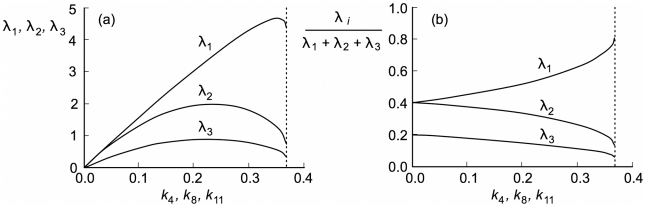
Contribution of the convex basis elements to the flux distribution at the steady state, for different values of the degradation rate constants defined within the region of bistability. (a) 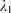, 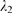 and 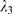 represent the contributions of the elements 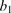, 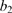 and 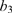 respectively. (b) The relative contributions of the three elements are illustrated over the same range of values of 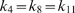.

To study the relative contributions of the basis elements to the steady-state flux distribution, 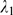, 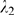 and 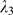 were evaluated from the numerical integration results for different values of the degradation rate constants (Fig. 8). The optimum operating rate value is obtained when 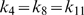 is in the range 0.2–0.3, rather closer to the conditions for bifurcation than those for equilibrium (Fig. 8a). The contribution of 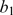 turns out to be around double that of 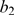 over most of the range. Nevertheless, as shown in Fig. 8b, as the degradation rate constants increase, the relative contributions of 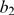 and 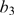 decrease steeply until the bifurcation point is reached for 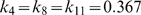. The rates of the replacement reactions, executed by these basis elements, then become incapable of withstanding the huge degradation rates, and the system collapses.

In the present model, the elementary flux modes coincide with the elements of the convex basis. Nevertheless, none of them is an enzyme-maintaining mode [23] because none of 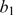, 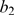 and 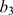 could indefinitely function alone, i.e. STU acting in 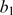 and 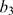 needs 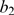 to be replaced but at the same time ST and SU in 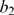 need the replacement function in 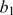 and 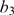, respectively (Fig. 7b). Thus, 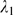, 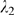 and 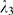 should all be greater than 0. This is the reason why the entire system constitutes an indivisible enzyme-maintaining mode.

## Discussion

A simple model of an (M,R)-system consisting of three catalytic cycles organized so that all catalysts are products of reactions within the system is able to establish and maintain a non-trivial steady state capable of resisting degradation of all the catalysts, provided that this degradation is not so fast that the catalysts are eliminated faster than they are regenerated. This model was originally proposed as a way of giving concrete expression to the abstract view of life embodied in Rosen's (M,R)-system s [1]. It does of course oversimplify some aspects of his analysis, but we consider that it is helpful for understanding the nature of his concept of closure to efficient causation. It shows that a small system in which all catalysts are produced internally can not only organize itself into a non-trivial steady state, but it can also recover from large perturbations, such as complete loss of a catalyst. In favorable conditions and with a large amount of time available, the system in stable steady state can create itself from essentially nothing — a few suitable reactants present in vanishingly small amounts. As mentioned above, no elementary flux mode in this model is independently capable of maintaining itself. We are conscious that this does not constitute a proof of the simplicity of this model. In fact the model in the form originally proposed [8] did not allow for degradation of ST (reaction 11 in Fig. 1), and in a sense, therefore, represented a simpler system. However, the inclusion of this decay process is more realistic when considering the capacity of ST to be driven to new processes of increasing complexity and thus the evolutionary potential of the model.

As our original model [8] was designed to be self-maintaining the demonstration here that it is indeed capable of self-maintenance confirms our prediction. The bistability that it also shows was not consciously designed. This leads to more complex dynamics, and the advantages of multistability for a living organism have been discussed previously [17], [18]. The model is composed of various interconnected reactions, and it can be decomposed into individual circuits according to either logical or stoichiometric criteria; it was, in fact, constructed logically, with interplay of three basic building blocks, as described in the Introduction. These three cycles have both structural and dynamic roles in the self-maintenance of the entire system, and they exert constraints on the conditions for a “living”, non-trivial steady state, as discussed already and illustrated by Fig. 8. We have checked that none of them exhibits bistability by itself, and the occurrence of multistationarity is a consequence of the combined action of all of them: no “living” steady state is achieved in the system if any reaction (other than a degradation step) of the model is eliminated.

As mentioned in the Introduction, a smaller system [19] than the one in Fig. 1 can show bistability: this was presented as the smallest chemical reaction system with bistability, but it is not a model of an organism because it includes no mechanism for regenerating the catalyst, and if this is lost, for whatever reason, no recovery is possible. We do not claim to have demonstrated that the model studied here is the simplest system capable of self-maintenance.

The simplicity of this robust self-maintaining system and its capacity to be easily seeded may allow us to regard it as a plausible prebiotic system. Specifically, the establishment of a reflexive autocatalysis, i.e. autocatalysis that results from the structure of the whole network rather than from specifically autocatalytic components, is a typical common feature of models that illustrate recent theories of the origin of life; for example, the “lipid-world” scenario [24] and the theory of autocatalytic sets of proteins [25] share this property. Although the chemical nature of the components in the system analyzed in this paper is not specified, its autocatalytic organization is sufficient to satisfy the definition of an autocatalytic set: STU catalyzes synthesis of SU and vice versa. Of course, the difficulty of spontaneously developing a realistic {STU, SU} dual set of molecules performing such a special task of autocatalysis is arguable, but no other simple model of organizational closure escapes this criticism either. In any case, the essential postulate is that acquisition of some kind of recursive autocatalytic network should have been a necessary step at the very beginning of prebiotic evolution, before the development of more complex infrabiological systems [26].

In this analysis we have effectively assumed that a primitive self-maintaining system has metabolism but does not have information processing, in other words a metabolism-first scenario for the origin of life. All of the principal current theories of life [1]–[5] incorporate metabolism, but only a minority [2], [5] explicitly incorporates storage of information; even the autocatalytic sets [4] treat RNA molecules only as catalysts, not as information stores. Particularly interesting is that this simple (M,R)-system shows functions that depend on the arrangement of elements in its intermediates: multiple components have the same composition but different functions, depending on the arrangement of their elements, e.g. SUSTU and STUSU are isomers with different activities, and the same is true of STUS and SUST. As the model is drawn, the structural differences are differences in *sequence*, suggesting sequence-dependent information storage even in a metabolism-first model of the origin of life: thus the borderline between replication-first and metabolism-first approaches to the origin of life may not be absolute. Indeed, this typical dichotomy may be blurred when considering simple organizational recursive systems in which the different chemical intermediates necessarily have parts of their structures in common.
